# Image processing for optical mapping

**DOI:** 10.1186/s13742-015-0096-z

**Published:** 2015-11-26

**Authors:** Prabu Ravindran, Aditya Gupta

**Affiliations:** Laboratory of Molecular and Computational Genomics, Department of Chemistry, Laboratory of Genetics and Biotechnology Center, University of Wisconsin, 425 Henry Mall, Madison, USA

**Keywords:** Optical mapping, Image processing, Skeletonization, Grouping, Tiling, Shortest path, Integrated fluorescence intensity

## Abstract

Optical Mapping is an established single-molecule, whole-genome analysis system, which has been used to gain a comprehensive understanding of genomic structure and to study structural variation of complex genomes. A critical component of Optical Mapping system is the image processing module, which extracts single molecule restriction maps from image datasets of immobilized, restriction digested and fluorescently stained large DNA molecules. In this review, we describe robust and efficient image processing techniques to process these massive datasets and extract accurate restriction maps in the presence of noise, ambiguity and confounding artifacts. We also highlight a few applications of the Optical Mapping system.

## Background

Optical Mapping [1–3] is a high-throughput, single-molecule system that generates ordered restriction maps (also called Rmaps) from high molecular weight genomic DNA molecules, ranging in size from 300 kilobases to a few megabases. The Rmaps are then used for the construction of genome-wide physical restriction maps using computational approaches, which provide insights into long range genome structure and genome variation. Optical mapping is made possible by the integration of many diverse components that draw from surface chemistry, microfluidics, fluorescence microscopy, image processing and other computational approaches. The physical maps generated using Optical Mapping have served as scaffolds to guide and/or validate DNA sequencing based genome assemblies [4–7]. More recently, Optical Mapping, because of its ability to resolve repeat rich and other low complexity genomic loci, has been used to identify structural polymorphisms in normal human genomes [3] and structural variants in disease-risk [8] and cancer genomes [9, 10].

**Fig. 1:**
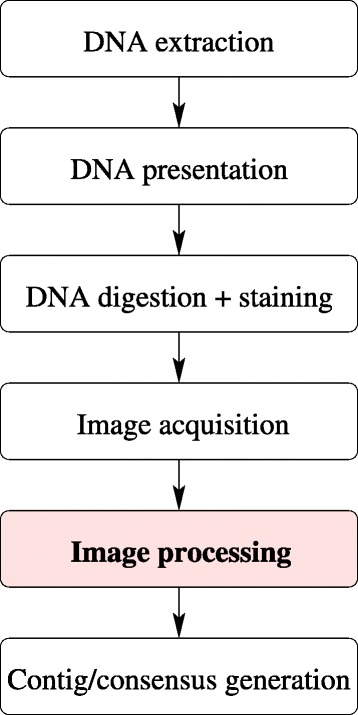
Schematic of the various steps in the optical mapping system

An outline of Optical Mapping is provided in Fig. 1. Here, we provide a brief description of the system. The first step in Optical Mapping is DNA extraction. Because high molecular weight DNA is required as a substrate, very gentle DNA extraction methods like liquid lysis of cell suspensions or preparation of DNA inserts [11] are commonly used. Next, DNA is presented on glass cover slips that are acid cleaned and derivatized with a mixture of aminosilanes. The derivatization process imparts a positive charge to glass surfaces, which allows DNA immobilization [5, 12–14]. DNA presentation is accomplished *via* capillary flow in microchannels, which are formed at the interface of derivatized glass surfaces adhered to a microfluidic device fabricated using soft lithography approaches [2]. Use of a microfluidic device allows for massively-parallel, high throughput deposition of single DNA molecules on derivatized glass surfaces. DNA presentation accomplishes two goals: elongation and immobilization. DNA elongation allows the imaging of molecular cleavage events once intact DNA molecules are digested using restriction endonucleases, and is an important requirement for generation of Rmaps. DNA immobilization serves to fix DNA in place, which is important to ensure that i) the linear order of DNA fragments from each DNA molecule is preserved; ii) the digested molecules can be imaged easily; and iii) the fragments generated after restriction digestion do not desorb and get lost before imaging. Both these steps, elongation and immobilization, are carefully controlled to ensure that the biochemical action of restriction endonucleases is preserved and that the DNA molecules are optimally stretched out to be able to generate useful Rmap data.

The elongated and immobilized DNA molecules are digested with a restriction endonuclease of choice. Upon digestion, the double-stranded DNA digestion sites present as gaps that are formed between fragments due to DNA relaxation at cut ends [1, 12]. Next, digested DNA is stained using intercalating fluorochrome YOYO-1 [13] and imaged using automated laser-illuminated epifluorescence microscopy systems [2, 15–17]. Custom in-house software allows automated imaging of an entire array of microchannels with very little setup time. Once the images have been collected, they are automatically processed using custom image processing software to generate Rmaps, which are obtained as ordered series of fragment sizes derived from digested single DNA molecules [2, 3]. Once a large dataset of Rmaps has been collected using the Optical Mapping system, a computational pipeline that uses Bayesian inference approaches [18] and cluster computing is used to assemble the Rmaps into genome-wide contigs and generate genome-wide consensus maps [3, 19–21].

The description above highlights that the image processing module acts as a filter/bridge within the Optical Mapping pipeline that extracts the useful essence, the Rmaps, from massive optical microscopy datasets. Image processing is a critical contributor to successful implementation of Optical Mapping and works in synchrony with the other components of the system. The image processing module is the central focus of this manuscript.

## Review

### Image processing methodology

**Fig. 2:**
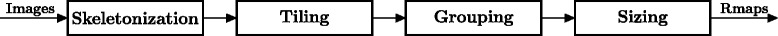
Schematic of the image processing module that extracts Rmaps from images

The goal of the image processing module is to accurately and robustly extract the Rmaps data from image datasets. An image processing module for Optical Mapping must provide the following capabilities (Fig. 2):

- Skeletonization: The single pixel centerline for each fragment is detected as a column ordered connected component or skeletal segment.
- Tiling: A single Rmap can span multiple images. In order to extract multi-frame Rmaps, a mosaic of images acquired from a single microchannel is created by aligning adjacent images using the skeletal segments.
- Grouping: The skeletal segments are grouped such that each group corresponds to fragments from the same DNA molecule.
- Sizing: Groups of skeletal segments that correspond to standards are detected; conversion factors for these skeletal segments are computed using integrated fluorescence intensity and the known size of fragments for the standards. The estimated conversion factor is applied to construct Rmaps (in kilobases) for genomic DNA molecules.

Different versions of image processing software for Optical Mapping have been implemented over the last two decades. During the early days of Optical Mapping useful map data was obtained using completely manual methods for detecting and sizing the fragments. Improvements to the image acquisition [15] and image processing software [16] culminated in the development of the semi-automatic Autovis system [5]. Lim and coworkers described Semi-Autovis in [5], where they used it to generate R-maps for the E. coli genome. As the name suggests, Semi-Autovis was a semi-automatic image processing system; it required user identification of the approximate location of suitable molecules. Once such locations were identified, Semi-Autovis handled skeletonization, grouping and sizing automatically. This system also dealt with crossing molecules, bright spots near molecules and other object imperfections, which was not possible with prior image processing systems. For E. coli, a total of 840 R-maps were collected (494 with XhoI; 346 with NheI), of which 471 were included in the final contigs (251 for XhoI and 220 for NheI), reflecting a contig rate of 56 %. Although Semi-Autovis was much faster than previous systems, there was clear need for a completely automated image processing system for Optical Mapping of larger genomes. PathFinder [2, 17] was the first fully featured, automated image processing system developed for Optical Mapping and was instrumental is making large scale Optical Mapping projects feasible. The image processing methodology detailed below is inspired by the techniques implemented in the PathFinder system.

### Skeletonization

**Fig. 3:**
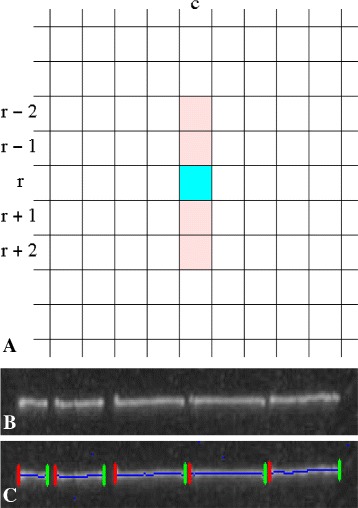
Skeletonization. **a** The local neighborhood in which the inequality constraints (1)-(4) are applied to detect skeletal pixels. **b** An example image patch. **c** Extracted skeleton in the example image patch. Skeletal pixels are shown in blue. Red and green lines represent the left and right endpoints of each skeletal segment

The first step extracts skeletal segments that correspond to the digestion induced fragments of DNA molecules. An image pixel *I*(*r*,*c*) is a skeletal pixel if *all* of the following conditions are satisfied:

(1) $$\begin{array}{*{20}l} I(r, c)~&\geq~I(r - 1, c)  \end{array} $$

(2) $$\begin{array}{*{20}l} I(r, c)~&\geq~I(r + 1, c)  \end{array} $$

(3) $$\begin{array}{*{20}l} I(r, c) - I(r - 2, c)~&>~\delta_{f}  \end{array} $$

(4) $$\begin{array}{*{20}l} I(r, c) - I(r + 2, c)~&>~\delta_{f}, \end{array} $$

where *I*(*r*,*c*) represents the image intensity at pixel coordinates (*r*,*c*) and *δ*_*f*_ is an user specified threshold that denotes expected falloff in gray intensity over two pixels. The local neighborhood used for the computation is shown in Fig. 3(a). The above constraints and geometry for the neighborhood are based on the facts that (i) the DNA molecules are deposited along the direction of flow in microchannels and (ii) the ideal fluorescence intensity profile falls off rapidly from the peak intensity, perpendicular to the deposited molecules. This physically motivated local computation results in an intuitive, efficient and robust direct gray scale skeletonization technique.

While the conditions in Eqs. 1 and 2 maintain connectedness of the skeletal pixels in a segment, skeletons that are not one pixel wide may also be produced. Hence, for each segment, the one pixel wide skeleton is extracted as the shortest path between the segment end points using Dijkstra’s algorithm [22]. The accurate localization of end points is aided by the increased intensity pixels due to coil relaxation at enzyme cleavage sites. An example extracted skeleton with end points is shown in Fig. 3(c).

### Tiling

The image acquisition system captures multiple overlapping images along each microchannel. Accordingly, long DNA molecules that span several frames are imaged, which necessitates tiling. As a linear stage is employed to acquire overlapping images, the geometric transformation that is used to model the tiling of adjacent images is a translation.

**Fig. 4:**
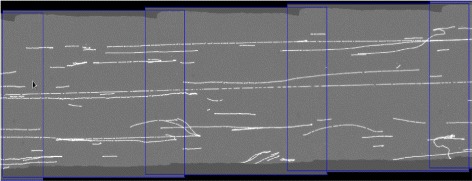
Tiling. Example of tiling three images from a microchannel. A horizontal span of about 140 microns is covered by each image. The blue outlines represent the image borders. The estimated tiling transformation provides the translation of frames to achieve this seamless mosaic, but the mosaic itself is not explicitly constructed. Estimating the tiling transformation is required to extract multi-frame Rmaps

The translation between adjacent frames is estimated using the left and right end points of the extracted skeletal segments as landmarks. Given two adjacent images, the translation that matches the maximum subset of landmarks from one image to the other is taken as the tiling transformation. As the amount of overlap between images is engineered into the acquisition process (typically 25 %), search for the tiling translation is localized to a small range of values. Having the tiling information between pairs of images is required to extract Rmaps that span multiple images. It is not required to explicitly create a single mosaic of the entire channel (see Fig. 4).

### Grouping

Skeletal segments that come from the same DNA molecule are incrementally grouped and column ordered using spatial proximity of skeletal segment end points and directional agreement with respect to fluid flow based deposition. Specifically, the grouping constraints are:

- A skeletal segment can belong to utmost one group.
- Adjacent skeletal segments in the same group have no overlap.
- For segments *S*_*l*_ and *S*_*r*_, the ordered grouping (*S*_*l*_,*S*_*r*_) is valid if *S*_*r*_ is the best segment to pair with *S*_*l*_ when “growing” *S*_*l*_ on its right *and* vice versa. When determining the best segment both spatial proximity and orientational similarity of the segments (*via* straight line fits) are used.

**Fig. 5:**
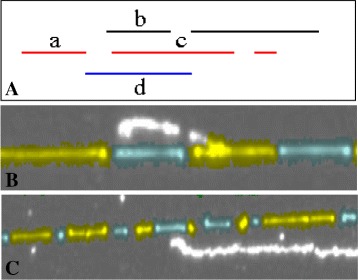
Grouping. **a** Segments *a* and *c* are grouped as this grouping provides the best continuity in terms of adhering to the constraints of spatial proximity and orientation similarity. The grouping (*a*,*b*) satisfies the spatial proximity constraint but in terms of orientation similarity, it is less optimal that the grouping (*a*,*c*). The grouping (*a*,*d*) is invalid as we require the segment *d* to be strictly non-overlapping with segment *a*. **b**, **c** Two examples of accurate grouping in the presence of distractors. The adjacent fragments are colored differently to aid visualization

The constraints and examples of grouping in the presence of distracting artifacts are depicted in Fig. 5.

### Sizing

The two main factors that influence fragment sizing are: (i) intensity fluctuations due to local variations in the elongation of the DNA molecule or staining, and (ii) regions of increased gray level intensity adjacent to enzyme cleavage sites due to coil relaxation. Fragment sizes obtained using integrated fluorescence values are robust to these local effects. In order to convert the integrated fluorescence values into kilobases, standards are used to used to estimate the conversion factor.

Within the grouped skeletal segments, groups that correspond to the standards are identified based on the expected number of fragments and their relative lengths. A mask that extends for two pixels on either side of the skeletal pixels is created and the fluorescence intensities in this region are summed to yield the integrated fluorescence values. The conversion factor *C*_*kb*_ that maps the integrated fluorescence values into kilobases is estimated (using the fragments of the standards) as the ratio:

(5) $$ C_{kb} = \frac{\text{size of fragment in kilobases}} {\text{integrated fluorescence value of fragment}}.  $$

**Fig. 6:**
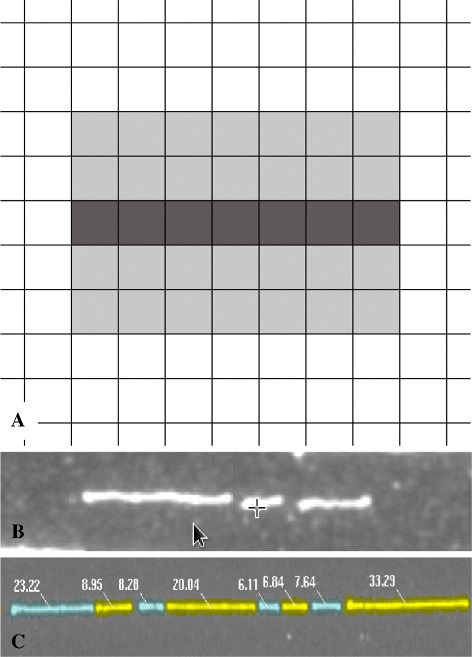
Sizing. **a** The two pixel wide mask used to compute the integrated fluorescence values for fragments. The darker grey pixels represent the skeletal pixels. Shown here is a fragment that spans 7 pixels. **b** An example three fragment, Bsu36I digested, Lambda DNA standard (≈48.5 kb) used to estimate *C*_*kb*_. The expected fragment sizes (in kilobases) are: 26.718, 7.601 and 14.183. Standards can be selected based on the experiment. **c** A portion of a Rmap with fragment sizes using the estimated *C*_*kb*_ to determine sizes in kilobases from integrated fluorescence values

The estimated *C*_*kb*_ is used to construct Rmaps from groups of ordered skeletal segments by converting integrated fluorescence values (computed using the same masks used for the standards) to kilobases (Fig. 6).

### Discussion

The skeletonization technique presented here robustly detects each fragment as a single skeletal segment. This technique can be easily adapted to other optical single molecule platforms such as nanocoding [23] and Irys [24] by evaluating the skeletonization conditions (Eqs. 1–4) in a direction perpendicular to the dominant direction of the presented molecules.

The spatial proximity and orientation similarity parameters that are used for grouping adjacent skeletal segments are empirically derived. For two skeletal segments to be grouped, we typically require spatial proximity to be less than 9 pixels (at 100 nm per pixel) and orientation difference to be less than 15 degrees. Higher enzyme restriction density can confound these thresholds as smaller fragments can “float” away and may not be ideally localized for Rmap grouping. In such cases ambiguities in grouping are handled using bioinformatics filters [25]. It should be noted that perfect handling of this situation is highly non-trivial; however when intact molecules (without restriction induced fragments) are localized in nanoslits ([23, 24, 26]), grouping becomes trivial.

Uncertainties in sizing are caused by variations in image intensities, ambiguities in localizing end points and distracting elements that can intersect molecules. The integrated fluorescence based sizing is highly resilient to the first two sources of uncertainties. Nearby and intersecting distractors are handled by “flagging” the affected fragment(s) in the Rmap and addressed using bioinformatics [25].

We highlight the effectiveness of the optical mapping system in providing detailed characterization of structural variants at the single molecule level using two exemplar large scale studies ([3, 10]). For the 4 human genomes that were studied in [3], over 95 % of fragments (≥ 10 kb) were within 10 % of their corresponding reference fragment size, indicating high accuracy in fragment sizing. Collectively for the four genomes, close to 27 % of all marked up molecules were assembled into contigs for final assemblies. In a more recent study that characterized a highly reorganized multiple myeloma genome [10], close to 29 % of all marked up molecules were assembled. We would like to stress that the performance metrics that we have mentioned encompass errors at the different stages of optical mapping, namely: DNA presentation, digestion, labeling, surface inconsistencies, imaging and image processing. While the effectiveness of the system as a whole is quantifiable (and ultimately what matters), it is still unclear how a stage-wise characterization of errors can be performed especially in the context of huge interesting genomes (the genome size regime in which optical mapping has the greatest impact).

The time taken to process the images from a single channel is typically faster than the time taken to collect the images. Hence we have not employed parallelization strategies for the image processing. Parallelization strategies will be highly attractive as the speed at which data collection improves. The skeletonization, tiling and sizing modules described in this paper can easily and trivially exploit data parallelism techniques.

## Applications

Fully automated image processing allowed for rapid analysis of DNA molecules deposited in microchannels, which helped us understand key physical characteristics of the deposition process (such as DNA elongation and deposition density along the microchannel) and design optimal operating parameters for Optical Mapping [2]. This enabled the generation of massive Rmap datasets, which facilitated high resolution analysis of genomes of various sizes.

Rmap assemblies provide long range structural information about the genome. Consequently, they generate a scaffold that can be used to verify or guide DNA sequencing based genome assemblies. Optical Mapping was first used to verify sequencing based chromosomal [27] and genome assemblies [4]. With an increase in throughput, it was used to generate physical assemblies to aid sequencing based genome assembly for many microbial genomes. These include some bacterial genomes like *Deinococcus radiodurans* [4], *Escherichia coli* O157:H7 [5], *Yersinia pestis* [28] and *Rhodobacter sphaeroides* 2.4.1 [29]. By comparing different bacterial strains to identify genomic differences, Optical Mapping was used for comparative genomics [17]. More recently, plant genomes like rice [6] and maize [7, 30] and normal [3] and cancer [9] human genomes have been mapped. These assemblies have helped in validation of sequencing based assemblies and have also provided high-resolution scaffolds for gap closure and for correcting sequencing based assembly errors [31].

In the past decade, advances in genome analysis methods have highlighted the widespread presence of structural changes in normal and disease-affected human genomes [32–34]. However, these variants have been found to be selectively enriched in segmentally duplicated and other low complexity regions of the genome [32, 35]. Because of the inability of short-read DNA sequencing data to uniquely differentiate these regions, true positives are difficult to discern in these regions. Additionally, false negative rates as high as 37 % have been reported [36], which could still be an underestimate. It is because of these reasons that different sequencing based structural variation calling algorithms show very little overlap [37]. Optical Mapping of human genomes has uncovered a wide array of structural variation in these genomes. Teague et al. identified thousands of structural polymorphisms, ranging in size from a few kilobases to megabases in a complete hydatidiform mole and three lymphoblast-derived cell lines [3]. The authors also identified many structural variants that could not be detected by other genomic analysis platforms. Later, Ray et al. studied tumor genomes from two oligodendroglioma patient samples, the first use of Optical Mapping to study a solid tumor genome, to reveal many somatic structural variants and copy number heterogeneity [9]. More recently, we integrated long-range structural variation analysis from Optical Mapping and short range variation analysis from DNA sequencing data to comprehensively characterize variation in a multiple myeloma genome at different stages of disease progression [10].

Many other genome analysis platforms have been developed in the recent years to understand long range genome structure and structural variation. BioNano Genomics Irys technology has been used to identify structural variants in human genomes [24]. Pacific Biosciences SMRT sequencing [38] and Oxford Nanopore Technologies sequencing [39] have increased the average read length from hundreds of bases to tens of kilobases. Although affected by significantly higher error rates when compared to their short read sequencing counterparts, these platforms can provide long-range sequencing information about genomes. Moving forward, developing computational methods and pipelines that integrate results from mapping- and sequencing-based platforms, or better, leverage raw datasets to improve sequencing pipelines, will help us learn more about whole genomes.

## Conclusions

The successful implementation of the automated image processing techniques described in this review has allowed the high resolution analysis of many complex genomes. It has also enabled the study of the physical characteristics of DNA deposition using microfuidic systems. In addition variants of the image processing techiques described in this review have been incorporated into the Nanocoding system, a higher resolution and more accurate successor to Optical Mapping [23, 26].
